# Programmatic Results of Integrating Systematic TB Screening Across Diverse Outpatient Health System Entry Points in the Democratic Republic of the Congo

**DOI:** 10.3390/tropicalmed11030083

**Published:** 2026-03-17

**Authors:** Romain Kibadi Lungoy, Jean Ngoy Kitenge, Nuccia Saleri, Stephane Mbuyi Tshikunga, Papy Pululu, Emmanuelle Papot, Corinne Simone Merle, Anna Scardigli, Jean Pierre Malemba Tshibuyi

**Affiliations:** 1 National Tuberculosis Programme, Kinshasa 10037, Democratic Republic of the Congo; ngoyjean89@yahoo.fr (J.N.K.); stephanembuyi7@gmail.com (S.M.T.); pululupapy@yahoo.fr (P.P.); malemba20.sept@gmail.com (J.P.M.T.); 2 The Global Fund to Fight AIDS, Tuberculosis and Malaria, 1211 Geneva, Switzerland; nuccia.saleri@gmail.com (N.S.); anna.scardigli@theglobalfund.org (A.S.); 3 The Special Programme for Research & Training in Tropical Diseases (TDR), World Health Organization, 1211 Geneva, Switzerland; papote@who.int (E.P.); merlec@who.int (C.S.M.)

**Keywords:** tuberculosis, integrated TB screening, program quality efficiency, number needed to screen, TB care cascade, health systems performance, gender disparities, programmatic surveillance

## Abstract

The Democratic Republic of the Congo faces a high tuberculosis (TB) burden. In 2022, 61% of an estimated 402,000 TB cases were reported (World Health Organization Global tuberculosis report). To enhance case detection, the national TB program (NTP) introduced a program quality and efficiency approach (PQE), integrating systematic TB screening into outpatient departments (OPDs). Observational data of the PQE on the TB care cascade (from screening to treatment) across 70 sites in Kinshasa that initiated PQE during the first quarter of 2023 are presented. Data were collected monthly and validated during supervision visits, and disaggregated by sex, healthcare facility type (public, private, or faith-based), facility level (primary or secondary), and OPD within each facility. In 2024, 639,464 individuals were consulted in various OPDs in the participating facilities, 57% of which were female. The median number needed to screen (NNS) was 22.1, with an interquartile range of [9.5–104.3]. There was a significantly lower NNS observed in general practice and human immunodeficiency virus departments. Throughout the TB care cascade, women were less likely than men to be screened, tested, or treated. These findings, to be interpreted within the context of Kinshasa pilot facilities, provide insights to the NTP for developing PQE implementation research aimed at understanding the reasons for these discrepancies and informing NTP scale-up at the national level.

## 1. Introduction

The Democratic Republic of the Congo (DRC) is among the top eight countries with the highest tuberculosis (TB) burden globally [1]. In 2022, the TB total yearly incidence was estimated at 402,000, while notifications of new and relapsed TB cases reported by the national TB program (NTP) reached 246,100, representing a TB treatment coverage of 61%, which remains below the 90% target [1]. Despite progress [1,2], a substantial gap persists in case detection, highlighting the need for innovative strategies to improve early diagnosis and linkage to care.

Alongside TB contact investigations and community-based active case findings, integrated screening models have been shown to improve TB detection rates and optimize resource use, particularly in high-burden settings—a key component of the World Health Organization (WHO) End TB strategy [3,4]. Recent studies have highlighted that embedding TB screening into routine health services could enhance TB case detection, treatment initiation, and successful treatment, with cost-effectiveness outcomes dependent on health system capacity and resource availability [5,6,7,8,9,10].

To address the TB treatment coverage gap in DRC, the NTP piloted several strategies to strengthen both active and passive TB screening, including the introduction of a program quality and efficiency approach (PQE) in 2022 with the financial support of The Global Fund. This new strategy in DRC aimed to strengthen TB case detection by integrating systematic screening within outpatient departments (OPDs) in various healthcare facilities in Kinshasa. The advantage of the PQE approach is to put the health facility staff at the center of the TB cascade analysis and plan to improve programmatic results. A screening questionnaire was based on the WHO four-symptom screen (W4SS) [11]. The NTP PQE surveillance team refined the training, material, and supervision based on initial feedback. In this stepped scale-up, the PQE approach was subsequently expanded to 70 additional facilities within Kinshasa, starting in 2023.

To describe the introduction of this approach, the programmatic data routinely collated in line with this new NTP recommendation—TB screening numbers and basic care cascade indicators, disaggregated by sex, healthcare facility type (public, private, or faith-based), facility level (primary or secondary), and OPDs within each facility—are presented here to discuss the overall observational data of the PQE in DRC.

## 2. Materials and Methods

**Design**—This article reports on routine NTP surveillance data relating to the PQE/W4SS strategy implemented on programmatic bases by the NTP of DRC since 2022. This report covers the period from 1 January 2023 to 31 December 2024, using a monitoring and evaluation approach.

**Setting and participants**—The 70 facilities were selected across Kinshasa, DRC, based on their historic TB notification, population at risk of TB in the catchment area, and the accessibility of TB services. A panel of secondary facilities (five General Hospitals) and primary healthcare centers (PHCs, 65) were chosen for this second wave of pilot across public, private, and faith-based facilities. Each selected facility had one to nine OPDs involved, among perinatal care consultation, school-age health consultation, diabetology, general medicine, internal medicine, nutrition services, pediatrics, emergency care, and human immunodeficiency virus (HIV) services; for a total of 42 entry points in General Hospitals and 412 in PHCs.

All individuals registering at one of these OPDs were administered the screening questionnaires as per NTP recommendations, whatever their age, if they (or their parents/guardians) were able to answer (these are referred to as ‘individuals eligible’).

**Intervention**—In 2022, the NTP conducted a situational analysis at the healthcare facility level to identify obstacles to TB diagnosis and treatment, engaging entry-point healthcare staff. Roles and responsibilities were clarified to intend to improve the quality of TB diagnosis and care, while strengthening the link between entry points, laboratory and TB focal points. The approach included initial training and mentorship on the W4SS steps and TB screening cascade data collection and analysis. All 70 sites were initiated during the first quarter of 2023.

The W4SS checked whether any individuals presenting at one of the selected OPDs had known contact with a TB patient or any of the following symptoms at that time: cough, fever, night sweats, or weight loss. In children, the clinical examination included checking for enlarged non-tender lymph nodes. Those identified as individuals with presumptive TB were referred for free diagnostic testing, microscopy, or Xpert^®^ MTB/RIF assay (Cepheid, Sunnyvale, CA, USA) [11]. During monthly review meetings, the entry-point staff present the number of people screened, identified as presumptive, and referred to the laboratory. The laboratory staff compare data on the number of people referred and number of people tested for TB. An example of gap identification early in the roll-out was the significant attrition between individuals with presumed TB during screening and those who actually completed the diagnostic test. By analyzing the care cascade during these monthly meetings, the team realized that it was necessary to strengthen communication about the fact that TB testing was free. They also clarified that even if patients could not afford the other prescribed tests, they could still receive the TB test at no cost. Implementing an improved patient-communication plan and establishing support to accompany individuals with presumptive TB to the laboratory helped reduce this attrition. The role of the TB/PQE focal point was to facilitate the entire process, up to result delivery and the decision to treat if the test was positive. Scheduled mentorship visits and monthly review meetings to monitor progress were organized by the PQE focal point at each health facility, at which point routine data quality assurance for monitoring and surveillance registers were conducted. If gaps in the cascade were identified during monthly review meetings, a simplified plan was collegially proposed at the health facility level.

**Variable data source**—Data on the TB care cascade were collected monthly in paper form by the TB/PQE focal point of each of the 70 pilot facilities. It focused on the number of individuals being screened with the W4SS as per NTP guidelines. Data of interest were the number of individuals registered at the OPD entry point (according to facility’s routine process and register, i.e., ‘eligible individuals’), number of ‘individuals screened’ with the W4SS (reported by healthcare workers at each OPD to the TB focal point), number of individuals with presumed TB (i.e., exhibiting one or more signs or symptoms outlined by the W4SS as described above, and who should be addressed to the TB focal point), number of individuals undergoing a TB test (microscopy or Xpert^®^ MTB/RIF, i.e., ‘individuals tested’), and the number of these individuals with a positive TB test (overall and less than 15 years old, i.e., ‘individuals with confirmed TB’), and of those, the number of individuals on TB treatment. Each of these variables were recorded by sex. Data were centralized by the NTP during the quarterly validation with the Kinshasa TB coordinator and transcribed into Excel^®^ spreadsheets.

**Study size and statistical methods**—The study included all NTP surveillance records available during the observation period (2023–2024); therefore, the sample size was determined by data availability.

The TB screening and care cascade are presented as numbers and frequencies.

The number needed to screen (NNS) to find one TB case was calculated as the inverse of the prevalence (ratio of individuals screened to individuals with confirmed TB) [12,13,14,15]. For these descriptive statistics, the NNS was presented as crude NNS, and summarized using median and interquartile range (IQR) due to its skewed distribution. The median per OPD was chosen to represent the typical performance of points of entry relative to each other, providing a basis for operational benchmarking across facilities and informing decisions on how to optimally scale up the PQE strategy in the future. Screening volumes are also presented and discussed to contextualize these comparisons.

All variables are presented overall and disaggregated by sex, healthcare facility type (public, private, or faith-based), facility level (primary or secondary), and OPDs within each facility. To facilitate result presentation, the authors decided to focus on 2024 and to present 2023 data in Supplementary Material Table S1. Simple comparisons were conducted using Chi square or Fisher’s exact test and risk ratio (RR) to explore gender differences, with 95% confidence intervals (CI 95%). The Kruskal–Wallis and pairwise Wilcoxon rank-sum tests were used to explore differences in NNS. Statistical analyses were performed with Stata 19^®^ (StataCorp. 2025. Stata Statistical Software: Release 19. College Station, TX, USA: StataCorp LLC).

**Ethics**—This analysis used anonymized programmatic data routinely collected by the NTP (Ministry of Health) for monitoring and reporting purposes based on the PQE plan and monitoring tools validated by Health national authorities. Ethical approval was not required for this secondary data analysis of non-identifiable aggregated surveillance data.

## 3. Results

During the year 2024, 639,464 individuals registered at one of the 454 OPD entry points. Females represented 57% of the population (364,408). The median NNS was 22.1 (IQR [9.5; 104.3]). Table 1 below shows the repartition of these OPDs by facility type and level. Faith-based PHCs represented the largest proportion of facilities offering TB services, often located alongside other frontline OPDs such as perinatal care, school-age health consultations, general medicine, and HIV services.

The TB screening cascade for males and females is shown in Table 2 below. Females had a significantly lower risk of presenting with presumed TB (around a third less risk; *p* < 0.001) or of being diagnosed with confirmed TB (−25% in 2024; *p* < 0.001) compared to males. In the other steps of the cascade, females had a significantly lower chance of being administered the W4SS questionnaire, of being tested, and of being treated compared to males (−2%, −8%, and −1%, respectively).

**Figure 1:**
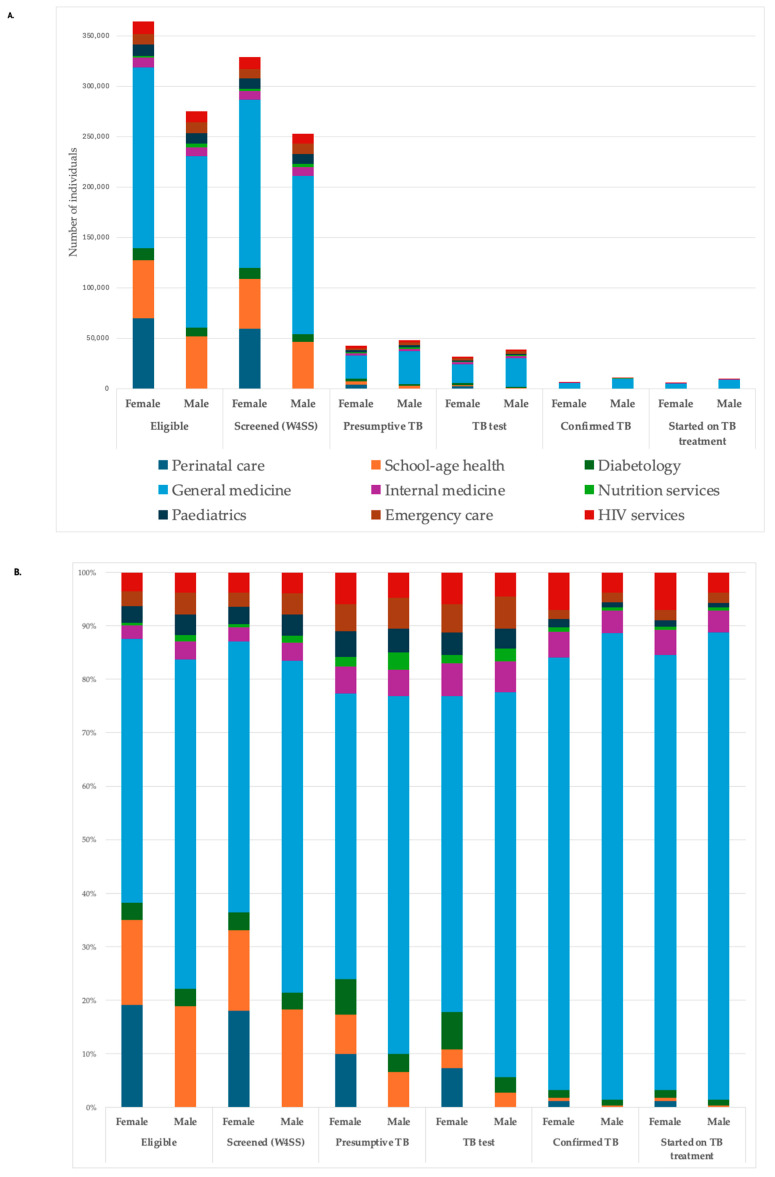
TB screening cascade by sex in the 70 sites in Kinshasa in 2024 (N = 639,464); (**A**) absolute numbers; (**B**) percentage.

The TB screening cascade by sex for 2024 is shown in Figure 1 below, both in absolute numbers and proportions by categories. Figure 1 highlights the weight of general medicine, internal medicine, emergency, and HIV OPDs in the overall cascade. In terms of absolute number, more females presented at any OPDs and thus were screened with the W4SS.

The TB cascade and NNS, in Table 3 below, is disaggregated by OPDs. It is shown that the perinatal care entry points had the highest number of individuals consulted, after those for general medicine and school-age health.

**Figure 2:**
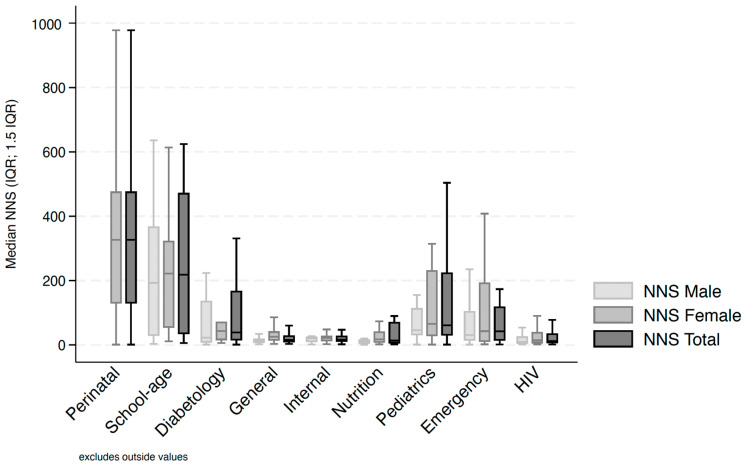
Number needed to screen to find one TB case (NNS) in 2024 disaggregated by sex and outpatient department (N = 639,464 across 454 OPDs). IQR: interquartile range.

The TB screening cascade in 2024 by entry point showed variability in terms of the number of individuals presenting at the different OPDs. The lowest NNS values were more significantly common for HIV services, general medicine, internal medicine, nutrition services, and emergency care, and to a lesser extent in pediatrics and diabetology. Figure 2 below highlights these observations. Discrepancies in the management of female versus male individuals were particularly significant in several steps (besides the number of presumed TB and confirmed TB cases). In diabetology, a larger proportion of women were tested (risk ratio = 1.12 (CI 95% [1.08–1.16])), whereas the opposite was observed in emergency care (risk ratio = 0.64 (CI 95% [0.62–0.67], *p* < 0.001)). In nutrition services, women were less likely to be initiated on TB treatment compared to men (risk ratio = 0.73, CI 95% [0.59–0.91], *p* = 0.003).

In 2024, the pilot facilities did not differ in terms of the NNS across levels, with a median of 20.4 (IQR [9.2–94]) for PHCs (585,304 individuals eligible) and 34.4 (IQR [18.2–169]) in General Hospitals (54,160 individuals eligible), *p* = 0.08. Faith-based structures had a significantly lower NNS, at 18.0 (IQR [7.9–54.0]; 330,192 individuals eligible), compared to both public facilities (30.5; IQR [12.5–133.5]; 188,325 individuals eligible) and private facilities (115.6; IQR [60.4–143.0]; 120,947 individuals eligible).

## 4. Discussion

Integrating systematic TB screening (W4SS) through a PQE approach in OPDs provided the NTP of DRC with an opportunity to monitor the TB screening cascade across 70 pilot facilities in Kinshasa. The choice of OPDs as entry points was appropriate, and those performing better aligned with the WHO’s TB screening recommendations for DRC [16]. While active case finding in contacts of patients with TB, people living with HIV and malnourished remains a priority, these findings underscore the value of screening the general population in high-burden settings during routine healthcare visits. The analysis revealed missed opportunities at multiple steps of the cascade, affecting both males and females, and in some instances more systematically affecting females—an area that warrants further investigation. The NNS can help identify OPDs that could be prioritized for continued implementation in the current funding context. Except for highly specialized services such as perinatal care and school-age health, most ODPs performed well.

The higher NNS among females was expected given the lower TB incidence in females, estimated at 176,000 in 2024 compared to 236,000 for males [2]. This pattern was reflected in our programmatic data in the proportion of presumed TB among individuals screened with the W4SS and the proportion of positive tests among individuals with presumed TB by sex.

Similar gender-related disparities have been reported in Nigeria. A retrospective review of their TB care cascades between 2018 and 2022 found that females had better access to screening but faced a higher testing gap compared to males, who were more likely to initiate treatment [17]. In our pilot, although more women were screened in terms of the absolute number, they were proportionally less likely to proceed to testing and treatment (linkage to care). These findings highlight the need to explore potential contextual factors contributing to missed opportunities and gender inequities. The programmatic data included in the analysis do not allow for the identification of specific biological characteristics, healthcare-seeking behaviors, patient flow within health services, or any bias in healthcare providers’ practices, which could explain the gender disparities in the results.

A recent systematic review and meta-analysis on perinatal TB screening showed that W4SS was low-cost and acceptable but poorly implemented, with an NNS of 138 (CI 95% [52–1429]) [10]. This entry point should also not be overlooked in DRC, even though its NNS was systematically the highest, as the number of eligible individuals is one of the largest, after general medicine. Performance review meetings led by the NTP could strengthen the adoption of the W4SS for women, and potentially their families visiting the service. They could also help clarify the care‑seeking journey of pregnant women, who may be referred to another OPD or may themselves seek care in other OPDs when presenting with symptoms not associated with their pregnancy. A recent review on pregnant and postpartum women [18] showed that this population faces a marked risk of TB yet remains under-represented in global estimates, highlighting the need for more targeted interventions and ensuring linkage to care, since testing and treatment initiation whenever needed remains critical for reducing disease burden.

TB detection in children, particularly younger ones, is challenging [19]. This report focused only on confirmed TB (with a positive test); thus, the results in this population should be interpreted cautiously. Although the use of Xpert^®^ MTB/RIF on stool samples has been scaled up in DRC since 2023, the concurrent use of WHO Treatment-Decision Algorithms (TDAs) for children below 10 years of age with presumptive pulmonary TB [20] is not reflected in these TB screening cascades, when the decision to treat was only based on clinical signs and symptoms and because TDAs were still in the early stages of national introduction in 2023–2024. Furthermore, unconfirmed TB cases (without bacteriological proof) may represent a significant proportion of diagnoses, which could mitigate the sex-disaggregated findings in the actual cascades.

PHCs did not perform differently from the secondary-level facilities, with more OPDs participating and a larger patient cohort. These findings should be refined, as task-shifting and decentralization seem good options for TB control, with a robust operationalization as to guarantee optimal management and linkage to care.

The limitations of the analysis stem from the reliance on aggregated programmatic data, lack of individual data, and absence of a comparator. We could not explore childhood TB in detail or assess NNS variations by diagnostic methods (microscopy versus Xpert^®^ MTB/RIF). Nonetheless, these pilot findings provide the NTP with a foundation for future implementation research aimed at improving effectiveness and assessing the acceptability, feasibility, and cost of the PQE approach for TB screening based on the W4SS. Such research could also help the NTP compare strategies according to site profiles, including the characteristics of the individuals within the facility catchment area, while accounting for patient volume. In addition, multivariate analyses could be used to refine and contextualize facilities’ OPDs performance by adjusting for factors such as diagnostic methods, patient demographics, and facility-level characteristics. Assessing acceptability and feasibility, and understanding the possible factors behind gender disparities, might help improve adoption and equitable utilization. Comparing TB notifications before and after PQE implementation could offer additional insights, in terms of the increase in TB detection, though changes may also reflect other concurrent NTP strategies to close the detection gap. Contextual factors such as the shortage of Xpert^®^ MTB/RIF cartridges and TB treatments in 2024 may also influence cascade performance, as well as the use of microscopy, which should be complemented by low-cost, low-complexity near-point-of-care diagnostic tests [21,22]. However, data reported for the WHO Global TB report between 2022 and 2024 showed an increase in TB diagnosis and treatment coverage from 61% to 70% [2].

Overall, these findings serve as a starting point for more in-depth investigation. While NNS values align with similar approaches in other high-burden countries, further data are needed to inform cost-effective recommendations.

## 5. Conclusions

The initial data suggest that the PQE approach led by the NTP in DRC is promising. We will continue to report and discuss the results with health facilities to jointly identify possible solutions to address the gaps highlighted. The current scaling-up to new provinces will be strengthened by addressing gender inequities and improving adoption and acceptability among healthcare workers and patients. Implementation research will be developed to understand the levers and barriers to optimal implementation of the W4SS, quality surveys will be conducted to assess the patients’ pathway for positive W4SS screening, and efforts will be made to better understand gender-specific barriers within the TB care cascade and the broader gender dynamics of TB treatment. Costing studies are essential, particularly in light of anticipated funding constraints. Continuous monitoring during the maintenance phase should be prioritized to ensure sustainability, refine strategies toward higher-performing OPDs, enhance patient-centered care, and incorporate quality improvement perspectives. New tools facilitating testing flow and linkage to care should be evaluated in this context, particularly in PHCs and OPDs with high patient volumes such as general medicine or perinatal care. All of these efforts align to support linkage to care, improve access to testing, and facilitate the timely initiation of treatment, thereby contributing to reduced disease burden.

## Figures and Tables

**Table 1:** Repartition of the entry points by type and level of facility (N = 454 outpatient departments across 70 facilities—5 General Hospitals and 65 primary healthcare centers).

OPDs	PHCs			General Hospital		Total (n; %)
	Faith-Based	Public	Private	Faith-Based	Public	
Perinatal care consultation	47	14	3	2	2	**68** (15.0)
School-age health consultation	45	14	3	2	3	**67** (14.8)
Diabetology	34	4	2	2	2	**44** (9.7)
General medicine	47	14	4	2	3	**70** (15.4)
Internal Medicine	14	8	3	2	3	**30** (6.6)
Nutrition services	21	10	1	2	3	**37** (8.1)
Pediatrics	21	11	3	2	2	**39** (8.6)
Emergency care	14	8	3	2	3	**30** (6.6)
HIV services	46	14	4	2	3	**69** (15.2)
**Total** (n; %)	**289** (63.7)	**97** (21.4)	**26** (5.7)	**18** (4.0)	**24** (5.3)	**454** (100.0)

**OPD**: outpatient department; **PHCs**: primary healthcare centers.

**Table 2:** TB screening cascade (conditional) for the 70 sites in 2024 (N = 639,464).

	Total n (% *)	Female n (% *)	Male n (% *)	Risk Ratio [CI 95%] Female vs. Male (*p*)
**Individuals eligible**	639,464	364,408	275,056	
**Individuals screened** with W4SS	582,250 (91.1)	329,075 (90.3)	253,175 (92.0)	0.98 [0.97–0.98] (*p* < 0.001)
**Individuals with presumptive TB**	90,862 (15.6)	42,727 (13.0)	48,095 (19.0)	0.68 [0.68–0.69] (*p* < 0.001)
**Individuals tested**				
**- Overall**	70,638 (77.7)	31,815 (74.5)	38,823 (80.7)	0.92 [0.92–0.93] (*p* < 0.001)
**- By type of test:**				
Microscopy	23,027 (32.6)	10,011 (31.5)	13,016 (33.5)	
Xpert^®^ MTB/RIF	47,611 (67.4)	21,804 (68.5)	25,807 (66.5)	
**Individuals with confirmed TB** (tested positive—yield)	17,981 (25.5)	6858 (21.6)	11,123 (28.7)	0.75 [0.73–0.77] (*p* < 0.001)
**Individuals started on TB treatment** (linkage to care)	16,275 (90.5)	6163 (89.9)	10,112 (90.9)	0.99 [0.98–0.99] (*p* = 0.02)
**Number needed to screen**				
**Crude NNS**	**32.4**	**48.0**	**22.8**	
Median for OPDs	22.1	27.0	14.4	
IQR	[9.5–104.3]	[12.4–93.1]	[6.9–39.7]	

**CI 95%**: 95% confidence interval; **IQR**: interquartile range; **NNS**: number needed to screen to find one TB case; **OPD**: outpatient department; **TB**: tuberculosis. * Proportion at each step of the cascade relative to the previous step (conditional), except for TB laboratory tests, where it is the proportion of microscopy or Xpert^®^ tests among all lab tests, and for children below 15 years old, where it is the proportion of children below 15 among individuals who tested positive.

**Table 3:** TB screening cascade (conditional) for the 70 pilot sites in 2024 disaggregated by outpatient departments (N = 639,464).

		OPDs (N = 454 Across 70 Sites)								
		Perinatal Care (n; % *)	School-Age Health (n; % *)	Diabetology (n; % *)	General Medicine (n; % *)	Internal Medicine (n; % *)	Nutrition Services (n; % *)	Pediatrics (n; % *)	Emergency Care (n; % *)	HIV Services (n; % *)
**Individuals eligible**	**Female**	69,571	57,848	11,881	179,596	9326	1998	11,322	10,038	12,828
	**Male**	NA	52,052	8677	169,650	8992	3583	10,444	11,145	10,513
	**Total**	NA	109,900	20,558	349,246	18,318	5581	21,766	21,183	23,341
**Individuals screened** with W4SS	**Female**	59,280 (85.2)	49,698 (85.9)	10,774 (90.7)	166,715 (92.8)	8938 (95.8)	1870 (93.6)	10,593 (93.6)	8861 (88.3)	12,346 (96.2)
	**Male**	NA	46,241 (88.8)	8001 (92.2)	156,858 (92.5)	8626 (95.9)	3418 (95.4)	9863 (94.4)	10,055 (90.2)	9950 (94.6)
	**Total**	NA	95,939	18,775	323,573	17,564	5288	20,456	18,916	22,296
**Individuals with presumptive TB**	**Female**	4246 (7.2)	3175 (6.4)	2818 (26.2)	22,846 (13.7)	2158 (24.1)	764 (40.9)	2057 (19.4)	2153 (24.3)	2550 (20.7)
	**Male**	NA	3162 (6.8)	1609 (20.1)	32,205 (20.5)	2366 (27.4)	1527 (44.7)	2153 (21.8)	2788 (27.7)	2285 (22.9)
	**Total**	NA	6337	4427	55,051	4524	2291	4210	4941	4835
**Individuals tested**	**Female**	2318 (54.6)	1131 (35.6)	2230 (79.1)	18,765 (82.1)	1947 (90.2)	508 (66.5)	1355 (65.9)	1668 (77.5)	1893 (74.2)
	**Male**	NA	1062 (33.6)	1136 (70.6)	27,942 (86.8)	2222 (93.9)	937 (61.4)	1426 (66.2)	2358 (84.6)	1740 (76.1)
	**Total**	NA	2193	3366	46,707	4169	1445	2781	4026	3633
**Individuals with confirmed TB** (yield)	**Female**	85 (3.7)	41 (3.6)	96 (4.3)	5543 (29.5)	329 (16.9)	57 (11.2)	106 (7.8)	117 (7.0)	484 (25.6)
	**Male**	NA NA	43 (4.0)	117 (10.3)	9703 (34.7)	459 (20.7)	73 (7.8)	106 (7.4)	208 (8.8)	414 (23.8)
	**Total**	NA	84	213	15,246	788	130	212	325	898
**Individuals started on TB treatment** (linkage)	**Female**	76 (89.4)	33 (80.5)	93 (96.9)	5009 (90.4)	291 (88.4)	36 (63.2)	76 (71.7)	113 (96.6)	436 (90.1)
	**Male**	NA	34 (79.1)	113 (96.6)	8828 (91.0)	416 (90.6)	63 (86.3)	85 (80.2)	196 (94.2)	377 (91.1)
	**Total**	NA	67	206	13,837	707	99	161	309	813
**Number needed to screen**	**Female** (median; IQR)	**326.5** [129.3–477.0]	**222.0** [53.6–323.4]	**43.1** [14.6–72.0]	**25.5** [13.1–42.4]	**22.0** [11.6–29.0]	**17.8** [7.5–42.0]	**65.6** [27.9–232.0]	**42.8** [10.0–193.65]	**14.4** [5.0–40.0]
	**Male** (median; IQR)	/	**193.0** [28.4–368.0]	**22.7** [7.0–137.0]	**11.0** [6.8–19.3]	**20.0** [8.6–26.0]	**10.6** [3.4–16.8]	**46.2** [30.1–114.5]	**31.1** [13.3–105.1]	**9.6** [4.0–26.5]
	**Total** (median; IQR)	/	**218.1** [34.1–472.5]	**39.1** [14.2–168.0]	**16.0** [8.5–29.7]	**16.4** [9.9–28.3]	**13.5** [4.8–71.6]	**61.1** [29.5–224.9]	**42.0** [13.7–119.4]	**12.0** [5.3–35.7]

**IQR**: interquartile range; **NA**: not applicable; **OPD**: outpatient department; **TB**: tuberculosis. * Proportion at each step of the cascade relative to the previous step (conditional), except for TB laboratory tests, where it is the proportion of microscopy or Xpert^®^ tests among all lab tests, and for children below 15 years old, where it is the proportion of children below 15 among individuals who tested positive.

## Data Availability

All data supporting the reported results can be found in the manuscript.

## Supplementary Materials

The following supporting information can be downloaded at https://www.mdpi.com/article/10.3390/tropicalmed11030083/s1: Table S1: TB screening cascade (conditional) for the 70 sites in 2023 (initiation year) and 2024 (maintenance year) (N = 1,194,798).

## Abbreviations

The following abbreviations are used in this manuscript:

CI 95%	95% confidence interval
DRC	Democratic Republic of the Congo
IQR	Interquartile range
NNS	Number needed to screen
NTP	National TB program
OPD	Outpatient department
PHC	Primary healthcare center
PQE	Program quality and efficiency
RR	Risk ratio
TB	Tuberculosis
W4SS	WHO four-symptom screening
WHO	World Health Organization
