# Supplementary material for: Programmatic Results of Integrating Systematic TB Screening Across Diverse Outpatient Health System Entry Points in the Democratic Republic of the Congo
Source: Trop Med Infect Dis. 2026 Mar 17;11(3):83. doi: 10.3390/tropicalmed11030083 (PMC13029867; doi:10.3390/tropicalmed11030083)
Supplement: Supplementary file 1 [file tropicalmed-11-00083-s001.zip › tropicalmed-4042971-supplementary.pdf]

Supplementary material

Supplementary material S1

Both yield and linkage to care have decreased between the initiation phase (2023) and maintenance phase (2024), though in terms of absolute numbers, more individuals were tested positive and linked to care while starting on TB treatment, thus notified, in 2024. Overall, yearly adoption significantly increased between induction and maintenance with 20% more chances to be actively screened (risk ration 0.80; CI95% [0.70-0.80]; p<0.001).

Table S1. TB screening cascade (conditional) for the 70 sites in 2023 (initiation year) and 2024 (maintenance year), (N=1,194,798).

	**2023** (initiation year)				**2024** (maintenance year)			
	**Total n (%*)**	**Female n (%*)**	**Male n (%*)**	**Risk ratio** [CI95%] Female vs. Male (p)	**Total n (%*)**	**Female n (%*)**	**Male n (%*)**	**Risk ratio** [CI95%] Female vs. Male (p)
**Individuals eligible**	555,334	312,360	242,974		639,464	364,408	275,056	
**Individuals screened** with W4SS	404,647 (72.9)	223,229 (71.5)	181,418 (74.7)	0.95 [0.95-0.96] (<0.001)	582,250 (91.1)	329,075 (90.3)	253,175 (92.0)	0.98 [0.97-0.98] (<0.001)
**Individuals with presumptive TB**	69,422 (17.2)	31,849 (14.3)	37,573 (20.7)	0.69 [0.68-0.70] (<0.001)	90,862 (15.6)	42,727 (13.0)	48,095 (19.0)	0.68 [0.68-0.69] (<0.001)
**Individuals tested**	51,829 (74.7)	22,319 (70.1)	29,510 (78.5)	0.89 [0.88-0.90] (p<0.001)	70,638 (77.7)	31,815 (74.5)	38,823 (80.7)	0.92 [0.92-0.93] (p<0.001)
- Microscopy	32,876 (63.4)	14,228 (63.7)	18,648 (63.2)		23,027 (32.6)	10,011 (31.5)	13,016 (33.5)	
- Xpert® MTB	18,953 (36.6)	8,091 (36.3)	10,862 (36.8)		47,611 (67.4)	21,804 (68.5)	25,807 (66.5)	
**Individuals with confirmed TB** (tested positive – yield)	16,560 (32.0)	6304 (28.2)	10,256 (34.8)	0.81 [0.79-0.83] (p<0.001)	17,981 (25.5)	6858 (21.6)	11,123 (28.7)	0.75 [0.73-0.77] (p<0.001)
- Including <15 years old	1576 (9.5)	739 (11.7)	837 (8.2)		2014 (11.2)	915 (13.3)	1099 (9.9)	
**Individuals started on TB treatment** (linkage to care)	16,008 (96.6)	6070 (96.3)	9938 (96.9)	0.99 [0.99-1.0] (p=0.04)	16,275 (90.5)	6163 (89.9)	10,112 (90.9)	0.99 [0.98-0.99] (p=0.02)
**Number needed to screen**								
**Crude NNS**	**24.4**	**35.4**	**17.7**		**32.4**	**48.0**	**22.8**	
Median for OPDs IQR	19.3 [7.2-60.7]	22.2 [7.7-78.1]	12.5 [6-37.6]		22.1 [9.5-104.3]	27.0 [12.4-93.1]	14.4 [6.9-39.7]	

CI95%: 95% confidence interval; **IQR**: interquartile range; **NNS**: number needed to screen to find one TB case; **OPD**: outpatient department; **TB**: tuberculosis.

* Proportion at each step of the cascade relative to the previous step (conditional), except for TB laboratory tests where it is the proportion of microscopy or Xpert® to the total number of lab tests, and for children below 15 years old where it is the proportion of children below 15 to the total number of individual tested positive.
